# Autophagy Paradox of Cancer: Role, Regulation, and Duality

**DOI:** 10.1155/2021/8832541

**Published:** 2021-02-11

**Authors:** Amit Kumar Verma, Prahalad Singh Bharti, Sahar Rafat, Deepti Bhatt, Yamini Goyal, Kamlesh Kumar Pandey, Sanjeev Ranjan, Saleh A. Almatroodi, Mohammed A. Alsahli, Arshad Husain Rahmani, Ahmad Almatroudi, Kapil Dev

**Affiliations:** ^1^Department of Biotechnology, Jamia Millia Islamia, New Delhi, India; ^2^Department of Biophysics, All India Institutes of Medical Sciences, New Delhi, India; ^3^Department of Anatomy, All India Institutes of Medical Sciences, New Delhi, India; ^4^Institute of Biomedicine, Cell and Tissue Imaging Unit, Finland; ^5^Department of Medical Laboratories, College of Applied Medical Science, Qassim University, Buraidah, Saudi Arabia

## Abstract

Autophagy, a catabolic process, degrades damaged and defective cellular materials through lysosomes, thus working as a recycling mechanism of the cell. It is an evolutionarily conserved and highly regulated process that plays an important role in maintaining cellular homeostasis. Autophagy is constitutively active at the basal level; however, it gets enhanced to meet cellular needs in various stress conditions. The process involves various autophagy-related genes that ultimately lead to the degradation of targeted cytosolic substrates. Many factors modulate both upstream and downstream autophagy pathways like nutritional status, energy level, growth factors, hypoxic conditions, and localization of p53. Any problem in executing autophagy can lead to various pathological conditions including neurodegeneration, aging, and cancer. In cancer, autophagy plays a contradictory role; it inhibits the formation of tumors, whereas, during advanced stages, autophagy promotes tumor progression. Besides, autophagy protects the tumor from various therapies by providing recycled nutrition and energy to the tumor cells. Autophagy is stimulated by tumor suppressor proteins, whereas it gets inhibited by oncogenes. Due to its dynamic and dual role in the pathogenesis of cancer, autophagy provides promising opportunities in developing novel and effective cancer therapies along with managing chemoresistant cancers. In this article, we summarize different strategies that can modulate autophagy in cancer to overcome the major obstacle, i.e., resistance developed in cancer to anticancer therapies.

## 1. Introduction

The term “autophagy” comes from “auto” and “phagy”, which means, respectively, “self” and “eating” in Greek. It is an evolutionary conserved catabolic process, critically required for the maintenance of cellular homeostasis, metabolism, and growth regulation. It is a self-degrading system in which autophagic substrates like damaged organelles, misfolded/aggregated proteins, and enzyme complexes are degraded. Autophagy acts as a quality control mechanism in cells. Physiologically, it is a strategy of survival under stress conditions by the renewal of the by-products such as amino acids, fatty acids, nucleotides, and carbohydrates. It is constitutively active at the basal level in healthy cells, under the tight regulation of the mammalian target of rapamycin (mTOR) and adenosine monophosphate-activated protein kinase (AMPK). The level gets significantly increased under stress conditions [1–3]. Apart from the degrading nature, it additionally takes part in biosynthetic and secretory processes [4]. It also has roles in development, differentiation [5, 6] organellar remodelling, quality control of organelles and proteins, genotoxic stress prevention, suppression of tumor, elimination of pathogen, immunity and inflammation regulation, maternal DNA inheritance, and programmed cell death [7, 8]. Malfunctioned autophagy is related to a variety of human maladies such as neurodegeneration, aging, and cancer [9]. Autophagy is also known as type II cell death mechanism. It has been found in some circumstances that autophagy can lead to cell death; for instance, autophagy shows association in degenerating neurons of Parkinson's and Alzheimer's patients, in MCF-7 cancer cell lines treated with 4-hydroxyxytamoxifen (selective estrogen receptor modulator), in the regression of corpus luteum [10], deterioration of Mullerian duct structure during male genital development [11], and involution of mammary and prostate glands [12–15]. But recent *in vivo* and *in vitro* studies suggested that autophagy acts as a major survival mechanism in response to various stresses like nutrient depletion, hypoxia, damaged organelles, accumulation of anomalous proteins, activated oncogenes, and deactivated tumor suppressor genes [16]. This review discusses the role, regulation of autophagy with a focus on cancer, and how autophagy is responsible in causing resistance to various cancer therapies. We summarized different strategies like pharmacological, RNA-based therapies and combinational drug therapies; conventional drugs with new formulations (nanoformulation) are inhibiting the autophagy and overcoming the autophagy-mediated tumor resistance against therapies. Besides, we also discussed about activators of autophagy as anticancer agents. Different drugs, natural products, or extracts along with siRNA combination enhance autophagy-mediated tumor cell death both in vivo and in vitro studies.

## 2. Autophagy Pathway

Autophagy, also referred to as macroautophagy, is an effective degradation mechanism. Substrates that have to be degraded are targeted to the double-membrane vesicles called autophagosomes which ultimately fuse with lysosomes. Autophagosome is a hallmark feature of autophagy. Autophagy is mediated by evolutionarily conserved genes called autophagy-related genes (Atg) and their encoded proteins (ATGs). Various Atg and their functions have been studied in yeast at different stages of autophagy [17]. There are more than 30 ATGs that have been discovered and about 17-20 ATGs take part in forming conjugation complexes that are necessary for the initiation of autophagy and autophagosome formation [18].

**Figure 1:**
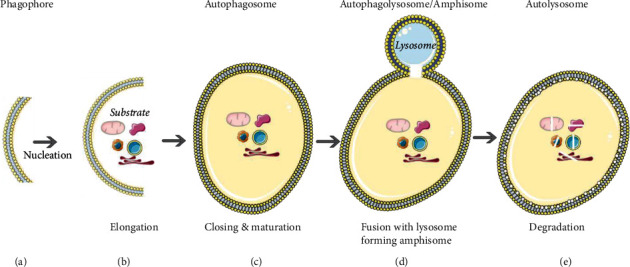
Stages in autophagy: (a) initiation—formation of double-membrane isolation membrane or phagophore; (b) nucleation and elongation—targeted autophagic substrate sequestration and elongation of the phagophore; (c) maturation—formation of autophagosome after the closure of phagophore with the entrapped substrate; (d) fusion—fusion of the mature autophagosome with lysosome forming autophagolysosome; (e) degradation—degradation of the substrate and inner membrane of autophagosome by lysosomal enzymes resulting in autolysosomes.

**Figure 2:**
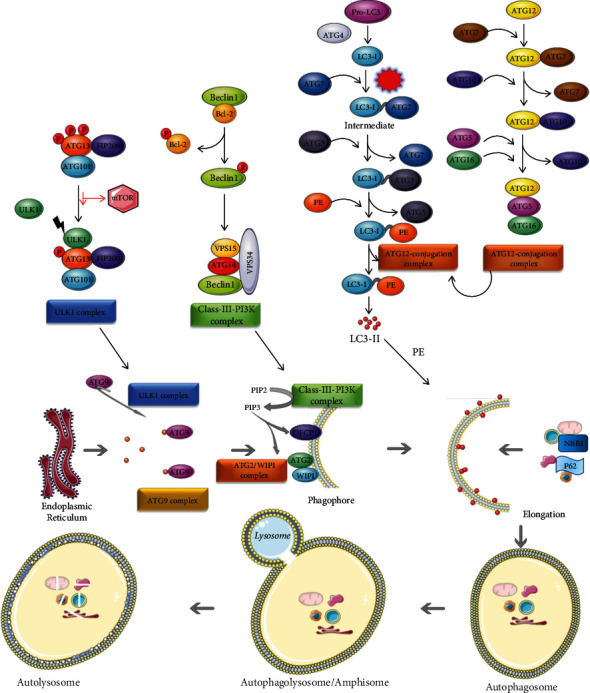
Autophagy pathway in mammalian cells: initiation of autophagy is dependent on 6 protein complexes: ULK1 complex, class III-PI3K (phosphoinositide 3-kinase) complex, ATG9 complex, ATG2-ATG18 complex, ATG8/LC3 complex, and ATG12 conjugation complex.

Autophagy process includes six steps—initiation, nucleation, elongation, maturation, fusion, and degradation (Figure 1) that are dependent on various protein complexes like Unc-51-like kinase 1 (ULK1 complex), phosphoinositide 3-kinase (class III- PI3K) complex, ATG9 complex, ATG2-ATG18 complex, ATG8/LC3 complex, and ATG12 conjugation complex (Figure 2).

### 2.1. ULK1 Complex

Induction of autophagy is dependent on the kinase activity of ULK1 [19, 20]. It is found in the inactive form if nutrients are readily available as it is dephosphorylated by the mammalian target of rapamycin (mTOR). During stress, a decrease in intracellular energy or an increase in the AMP/ATP ratio under low energy level activates adenosine monophosphate kinase (AMPK) which acts as a metabolic sensor by regulating glucose and lipid metabolism. [19, 21, 22]. AMPK-mediated deactivation of mTOR and phosphorylation of ULK1 lead to the activation of the ULK1 complex which consists of ULK1/ATG1, ATG13, FIP200/ATG17 (FAK-family kinase-interacting protein), and ATG101. ATG13 gets dephosphorylated (highly phosphorylated under nutrient-rich conditions), which enables the binding of ULK1 and increases its kinase activity.

### 2.2. Class III-PI3K (Phosphoinositide 3-Kinase) Complex

The PI3K-III complex containing Beclin-1/ATG6, ATG14, VPS34, and VPS15 (vacuolar protein sorting 34 and 15) is activated by ULK1 complex which is also important in the formation of isolation membrane or phagophore. VPS34 and VPS15 are class III-PI3 kinases, and VPS34 acts as a catalytic subunit, whereas VPS15 acts as a regulatory subunit in this complex. This complex phosphorylates PIP2 (phosphatidylinositol diphosphate) to PIP3 (phosphatidylinositol triphosphate) which is a prerequisite for the initiation and nucleation processes.

#### 2.2.1. ATG9 and ATG2-ATG18 Complexes

Increase in the PIP3 concentration leads to the recruitment of other proteins such as DFCP1 (double FYVE domain-containing protein 1), WIPI/ATG18 (WD-repeat domain phosphoinositide-interacting protein 1), ATG2, and ATG9 at the phagophore formation site [23]. During phagophore formation, endoplasmic reticulum (in the form of omegasomes) works as a membrane source in the presence of DFCP1 and ATG2-WIPI/ATG18 complex [24–26], while ATG9 mediates the use of membrane vesicles from the endoplasmic reticulum, plasma membrane, and mitochondria. In plasma membrane, ATG16L and heavy chain clathrin interaction is required for the formation of autophagosome precursor [27, 28], whereas, in mitochondria, ATG5 and LC3 localization to the outer membrane of mitochondria is required, which serves as a cornerstone for phagophore formation [29].

### 2.3. ATG8/LC3 and ATG12 Conjugation Complexes

Another protein that is important for preautophagosome elongation and maturation is MAP1LC3 (microtubule-associated proteins 1A/1B light chain 3) or LC3/Atg8. LC3 is found in cells in the inactive form, i.e., proLC3 which is converted to LC3-I by ATG4 (a cysteine protease). Along with LC3, ATG12 conjugation complex (ATG12-ATG5-ATG16) (E3-like protein) plays an important role in elongation and maturation of autophagosomal membrane as it helps in recruitment and conversion of LC3-I to LC3-II by adding phosphatidylethanolamine (PE) with the help of ATG7 (E1-like protein) and ATG3 (E2-like protein). This LC-II is recruited to the autophagosomal membrane through its lipid moiety and is not freely available in the cytoplasm like LC-I. When LC3-I is converted to LC3-II, autophagosome is elongated (marker to monitor autophagy) [30] and it becomes a vesicle that is called a mature autophagosome [31]. After completion, LC3 remains bound to the lumina. During nucleation and elongation, some adaptor proteins play important roles in cargo selection which is LC3-dependent. Ubiquitin-binding protein p62/sequestosome-1 (p62/SQSTM1), neighbor of BRCA1 (NBR1), nuclear dot protein 52 kD (NDP52), and optineurin transport their cargos to nucleation site by their LC3-interacting region (LIRs) or ATG8-interacting motifs (AIMs) which facilitate cargo selection as well as selective autophagy. Once the protein recruitment and formation of autophagosome are completed, the mature autophagosome with its contents fuses with lysosomes via sets of protein families: soluble N-ethylmaleimide-sensitive factor attachment protein receptors (SNAREs), including syntaxin-17 (STX17), synaptosomal-associated protein 29 (SNAP29), and vesicle-associated membrane protein 8 (VAMP8) [32]; Ras-related protein Rab-7 (Rab7); and HOPS (the homotypic fusion and protein sorting-tethering complex) [33]. After the fusion of the autophagosome with the lysosome, it becomes autolysosome. The autophagosome releases its content into the lysosome, and those substrates are degraded in the acidic environment of the lysosome by particular proteases (cathepsins B and L). “Alternative macroautophagy” has been introduced which is independent of LC-II, ATG5, and ATG7, but is critically dependent on ULK1 and Beclin1. It is debated whether it is a different form of autophagy [34]. At first, autophagy was believed to be a nonspecific degradative process. But now, many selective pathways have been demonstrated and are named according to the particular substrate that is degraded. For instance, for mitochondria, it is called mitophagy, for ferritin, it is called ferritinophagy, for ER, it is reticulophagy, for bacteria, it is xenophagy, etc. [35, 36].

## 3. Regulation of Autophagy

Nutritional status, energy level, growth factors, insulin ER stress, SOS (response when cells are exposed to stress causing DNA damage and cell cycle arrest), and other signals modulate the autophagy process by various target proteins. Of all target proteins, mTOR, also known as FRAP1, is a master regulator that negatively regulates autophagy [37, 38]. It consists of two complexes—mTORC1 (mTOR complex 1) and mTORC2 (mTOR complex 2)—out of which, mTORC1 is sensitive to rapamycin. Rapamycin (sirolimus) is a mTOR inhibitor extracted from bacterium *Streptomyces hygroscopicus.*

**Figure 3:**
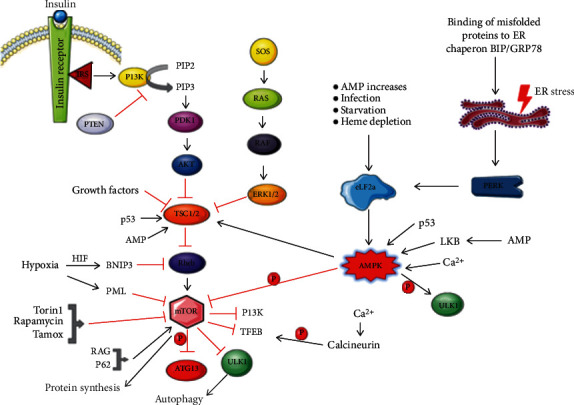
Regulation of autophagy: binding of growth factors to its receptor (tyrosine kinase) gets activated leads to the activation of PIP3K which phosphorylates PIP2 to PIP3 and recruits protein kinases PDK1 and AKT to inactivate TSC and RHEB. Activated GTPase Rheb activates mTOR which inhibits by phosphorylation of downstream targets of autophagy pathway like Beclin1 and Atg13 and transcription factors like TFEB by mTORC1 in nutrient-rich condition and inhibits autophagy. Other signals like SOS, AMP increase, starvation, and ER stress activate their signal cascade and regulate the PI3K-mTOR axis components.

In nutrient-rich conditions, mTORC1 hinders autophagosome formation and thus suppresses autophagy [39–42]. Upstream of mTORC1, growth factor receptors, and tyrosine kinase receptors get phosphorylated and activated which leads to the activation of PIP3K either directly or indirectly via adapters such as GAB2 [growth factor receptor-bound 2- (GRB2-) associated-binding protein 2] and insulin receptor substrate 1 (IRS1) [43, 44]. Activated PI3K converts PIP2 to PIP3 and recruits protein kinase 3-phosphoinositide-dependent protein kinase-1 (PDK1) and RAC-alpha serine/threonine-protein kinase1 (AKT1). AKT1 gets activated by PDK1 which inhibits heterodimerization of tuberous sclerosis 1 (TSC1) and TSC2, resulting in inhibiting Ras homolog enriched in brain (Rheb) and hence activation of its GTPase activity. GTP-Rheb activates mTOR which leads to autophagy suppression. Phosphatase and tensin homolog (PTEN) is a tumor suppressor which dephosphorylates PIP3 to PIP2 and counterbalances the whole cascade (Figure 3) [45]. Loss of tumor suppressors like liver kinase B1 (LKB1), promyelocytic leukemia protein (PML), PTEN, and TSC1/2 or gain in function mutations can activate mTOR, thus inhibiting autophagy, whereas cellular stress and mTOR inhibitors like rapamycin, Torin 1, and Tamox downregulate or inhibit mTOR and induce autophagy [46, 47]. Another mTOR complex mTORC2 is involved in the activation and phosphorylation of AKT1 [8, 48, 49]. 6-TG (6-thioguanine) induces autophagy and requires activation of mTOR [50]. So, it is not universal that mTOR activity is inversely proportional to autophagy.

Hypoxia (low oxygen status which can be present in cells in poorly vascularized regions) activates autophagy through hypoxia-inducible factor- (HIF-) dependent and hypoxia-inducible factor- (HIF-) independent pathways [16, 51, 52]. The HIF-dependent pathway involves selective autophagy mediated by HIF1 and its target Bcl-2 nineteen-kilodalton-interacting protein 3 (BNIP3) [53, 54]. They are affected by the concentration of oxygen and some growth factors which lead to modulation in the regulation of erythropoiesis, angiogenesis, metabolism, pH regulation, cell migration, and tumor invasion [53] by increases in the expression of angiogenic factors, such as vascular endothelial growth factor (VEGF), platelet-derived growth factor (PDGF), and nitric oxide synthase (NOS). BNIP3 activates Beclin1 by inhibiting Bcl-2 [52]. So, both HIF and BNIP3 are required for prosurvival hypoxia-induced autophagy in normal as well as in cancer cells [53–55]. Also, it has been seen that misfolded proteins can also regulate autophagy by binding itself to ER chaperone-binding immunoglobulin protein (BiP)/GRP78 and release three ER membrane-associated proteins: PKR-like eIF2a kinase (PERK), activating transcription factor-6 (ATF6), and inositol-requiring enzyme 1 (IRE1). PERK and ATF6 induce autophagy, whereas IRE1 inhibits [56].

### 3.1. Transcriptional Regulation of Autophagy

**Figure 4:**
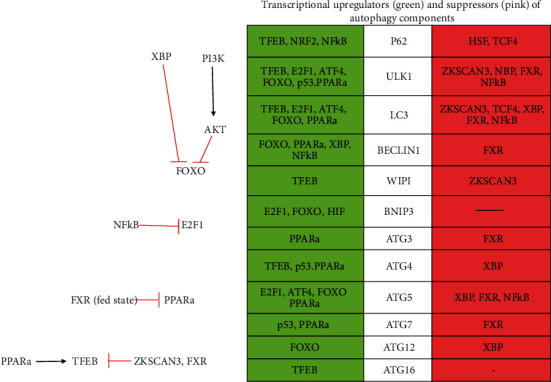
Transcriptional factors regulating autophagy [62].

There are some transcriptional factors which act as upregulators or downregulators of autophagy. For example, transcription factor EB (TFEB), E2F transcription factor 1 (E2F1), activating transcription factor 4 (ATF4), Forkhead box O3 (FOXO3), nuclear factor erythroid-derived 2-like 2 (NRF2/NFE2L2), hypoxia-induced factor (HIF), p53, and peroxisome proliferator-activated receptor *α* (PPAR*α*) are upregulators of autophagy, whereas zinc finger protein with KRAB and SCAN domains 3 (ZKSCAN3), heat shock factor (HSF), transcription factor 4 (TCF4), X-box binding protein 1 (XBP1), farnesoid X receptor (FXR), and nuclear factor kappa-light-chain-enhancer of activated B cells (NF-*κ*B) are suppressors of autophagy (Figure 4).

TFEB is the prime regulator of lysosomal biogenesis and gets activated by dephosphorylation during starvation which allows it to enter the nucleus to induce transcription of its targets such as Atg4, Atg16, LC3, p62, WIPI proteins, ULK1, and some cathepsins. [57–59]. E2F1 is involved in stress-related mechanisms and is inhibited by NF-*κ*B; it upregulates BNIP3, ULK1, LC3, and Atg5 and helps in the preautophagosome initiation [60–63]. ATF4 is upregulated by itself and activated by severe hypoxia; it enters into the nucleus and upregulates autophagy machinery components ULK1, LC3, and Atg5 [62, 64, 65]. FOXO3 is inhibited by AKT (which is activated by PI3K). Activated FOXO (suppressed AKT and PI3K) enters the nucleus and activates Atg4, Atg2, Atg5, Atg12, Beclin1, LC3, and ULK1 [62, 66, 67]. NRF2 is regulated by amino acid content and nutrient signaling and upregulates p62. HIF is activated by mild hypoxia, whereas ATF4 is activated during severe hypoxic conditions [53, 54]. p53 comes into picture during DNA damage; it upregulates Atg4, Atg7, and ULK1 [62, 68, 69]. PPAR*α* is activated during starvation, whereas, in the fed state, it is suppressed by FXR. PPAR*α* induces Atg3, Atg5, Atg7, Beclin1, LC3, TFEB, and ULK1 [70–72].

ZKSCAN3 is a negative and major regulator of TFEB. In a fed state, it enters the nucleus and binds to autophagy machinery target genes such as ULK1, LC3, and WIPI proteins [62, 73]. HSF is opposite of NFR2 as it suppresses p62 [74]. TCF4 is regulated by *β*-catenin. TCF4 suppresses p62 when *β*-catenin binds to TCF4. But when LC3 binds to *β*-catenin, it leads to proteasomal degradation. So, *β*-catenin suppresses TCF4. XBP1 is activated by ER stress and enters the nucleus. It has a dual function by upregulating Beclin1 and suppressing FOXO3 which in turn suppresses Atg4, Atg5, Atg12, LC3, and ULK1 [62, 75]. FXR is a negative transcriptional repressor of PPAR*α* and it inhibits genes of Atg3, Atg5, Atg7, Beclin1, L3, ULK1, and TFEB. NF-*κ*B has dual effects; it enters the nucleus and upregulates Beclin1 and p62; on the other hand, it also inhibits E2F1, i.e., inhibits Atg5, LC3, and ULK1 [22, 62, 63, 76–78].

During starvation, calcium ions are released into the cytoplasm from lysosome and activate a phosphatase calcineurin which removes phosphate from TFEB (phosphorylated by mTOR in a fed state) and gets activated. During starvation, ZKSCAN and FXR are kicked out of the nucleus which allows TFEB and PPAR*α* to enter into the nucleus, bind to its targets, and induce autophagy by induction of many autophagy proteins. During starvation, c-Jun also gets translocated into the nucleus which induces expression of ANXA2 (Annexin A2) which is important for increased secure autophagic trafficking [62, 79].

### 3.2. Regulation of Autophagy by p53

p53 is the “guardian” of cell integrity. It is a tumor suppressor protein and is mutated in more than 50% of the cancers [80]. It is reported in almost every type of cancer ranging from 10% in hematopoietic malignancy [81], 20-40% or more in breast cancer, colorectal cancer [82], and other type of cancers [83], and 50-60% in esophageal carcinoma to approx. 100% in high-grade serous carcinoma of ovary [84]. Depending upon the stimuli and subcellular localization, it can function as a positive as well as a negative regulator of autophagy. Nuclear p53 acts as an activator of autophagy in a transcription-dependent or transcription-independent manner. AMPK gets activated by stress-activated p53 which upregulates autophagy by downregulating the mTOR pathway. p53 also induces activation of the autophagy pathway by upregulating damage-regulated autophagy modulators (DRAMs), Sestrin 1 and 2, and death-associated protein kinase 1 (DAPK1). DRAM (a lysosomal protein) stimulates autophagy at various stages of the autophagic process. DAPK-1 stimulates autophagy by inhibiting antiautophagic microtubule-associated protein 1B (MAP1B) protein (an LC3 interactor). AMPK increases level of NAD^+^- and NAD^+^-dependent activity deacetylase Sirt1, which plays an important role in stimulation of autophagy by numerous ATG activations and deacetylations [85]. Cordani et al. reported that mutant p53 substantially counteracts the autophagic vesicle formation and fusion of these vesicles with lysosomes through the suppression of several important autophagy-related enzymes and proteins such as DRAM1, BECN1, and AMPK [86]. Nuclear p53 also releases Beclin1 via phosphorylation from the sequestration of Bcl-2/Bcl-xL/Mcl-1 [87]. It is also observed that deficiency of Beclin 1 is associated with deficiency in tumor suppressor p53, causing malignancies in BECN1-deficient mice [87–90]. Furthermore, p53 can also induce autophagy via transactivation of the p14ARF tumor suppressor (or p19ARF in mouse, or also known simply as ARF), which can stabilize p53 by inhibiting Mdm2 (E3 ubiquitin ligase) and subsequently inhibiting the proteasome-mediated degradation of p53 [91–94].

**Figure 5:**
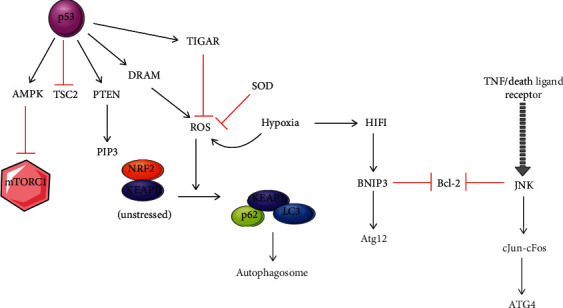
Regulation of autophagy mediated by p53: depending upon p53 subcellular localization, it plays a dual role in the process of autophagy. Nuclear p53 activates AMPK, inhibits mTOR, and induces autophagy. It was also induced by proautophagic modulators DRAM and PTEN. The inhibition of Bcl-2 leads to the release of Beclin 1 and activates autophagy, whereas in cytoplasmic p53, autophagy inhibition is associated with TIGAR.

However, cytoplasmic p53 acts as an inhibitor of autophagy. It targets TP53-induced glycolysis and apoptosis regulator (TIGAR) gene that downregulates glycolysis and suppresses ROS, rather than via the mTOR pathway (Figure 5) [90, 95]. Tasdemir et al. and Sui et al. have reported that cytoplasmic p53 directly interacts with FIP200, thus regulating the crucial initiation step of autophagy [80, 90]. To induce autophagy, it is required to degrade cytoplasmic p53. p53^−/−^ cells do not show any autophagic response when transfected with p53 protein in the cytoplasm. It is due to the lack of nuclear p53 [80]. This dual role is not exclusive to p53; potent oncogene Ras also has been reported to portray this dual role [96–98].

Ataxia telangiectasia mutated (ATM) (a sensor of cellular or DNA damage during the cell cycle) and high mobility group box 1 (HMGB1) (an immune modulator) also have been discovered as controllers of autophagy by regulating TSC2/mTORC1 signaling axis and interaction with Beclin1, respectively [99, 100].

## 4. Autophagy in Cancer

Cancer is a multifactorial disease characterized by uncontrolled cell division and can develop an obtrusive phenotype that is caused by genetic mutations, epigenetic alterations, and environmental factors that change the expression or function of the encoded products and behavioural factors [16]. GLOBOCAN (2018) estimated about 9.55 million deaths every year [101]. In India, about 0.7 to 0.81 million cancer-related deaths are estimated [102]. Cancer uses a variety of sources to meet the needs for unrestricted proliferation. It shifts towards anabolic metabolism and increases the Warburg effect (aerobic glycolysis) [103]. There are increasing evidence that autophagy regulates cancer cell's life. Cancer is one of the major maladies that is linked to dysregulation of autophagy. Autophagy deficiency prompts oncogenic mutations, damaged organelles, and macromolecule accumulation hence inducing oxidative stress, chromatin instability, DNA damage, and tumor susceptibility [104–106].

Tumor cells have more reliance on autophagy for survival as compared to normal cells due to their rapid proliferative ability in the nutrient-deprived environment caused by them. Through the autophagy recycling process, cancer cells overcome metabolic stress during its rapid proliferation. Dysregulated autophagy has implications in both well-being and sickness, specifically in cancer. Autophagy plays a dichotomous job in cancer by restraining tumor commencement (as a tumor suppressor); on the other hand, it is supporting tumor progression [107]. A few key autophagic regulators and their related pathways which play important roles in the regulation of autophagy in cancer include the Beclin1 interactome, Ras-Raf-MAPK pathway, PIR-Akt-mTOR pathway, and TP53 signaling. Several signaling pathways and molecules may modulate the autophagy in cancer, and these agents may act as potential therapeutic targets in treatment of cancer [108].

### 4.1. Duality of Autophagy in Cancer

A key question is how autophagy plays a dual role in the regulation of cancer. It prevents the development of the cancerous cells; on the other hand, it behaves as a key mechanism for the survival of the cancer cells. It was initially thought that autophagy has a tumor-suppressive role in normal cells by constraining inflammation, tissue damage, and prevention from senescence induced by an oncogene, scavenging damaged organelles and macromolecules, preventing the accumulation of radicles which are toxic and cause instability of the genome, and restricting the metastasis of cancer cells [8]. Also, by limiting tumor necrosis and oncogene-induced increase in senescence during an early stage of metastasis of cancer can act as a metastasis suppressor.

However, autophagy has also been implicated in benefits of cancer cells by either of the two mechanisms: one is the mutation which leads to activation of oncogenes (e.g., PIK3CA Ras, RHEB, and AKT—autophagy inhibitors) and the second being mutations that result in inactivation of tumor suppressor genes (e.g., PTEN AMPK, LKB1, and TSC1/2—autophagy inducers) [9]. The initiation of neoplasia in various genetically engineered mouse models (GEMMs) has also been reported by deleting the autophagy genes for studying autophagy deficiency in cancer. Paradoxically, autophagy can supply nutrients to cancers and, in turn, enhances its survival. In the advanced stage of metastasis of cancer, it promotes ECM disengagement of a metastatic cancer cell to a distinct site and also helps to enter the cell into dormancy if the cell fails to set up a contact with ECM [109]. Additionally, as a response to therapy or to confer resistance to radiations and chemotherapy, autophagy gets frequently upregulated and protects cancer cells from apoptosis [8, 16, 110, 111].

#### 4.1.1. Autophagy as a Tumor Suppressor

**Figure 6:**
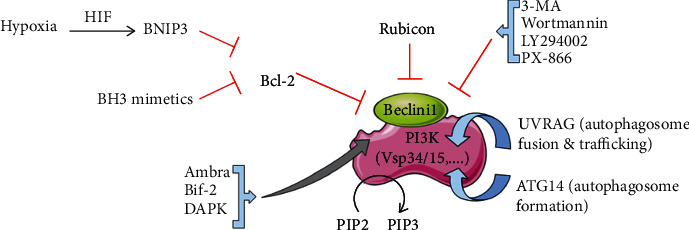
Regulation of Beclin1 and its associated protein activity. Beclin1 phosphorylates PIP2 to PIP3. Interaction of Beclin1 with Bcl-2 inhibits its activity. Ambra1, Bif-2, and DAPK induce autophagy by disrupting the interaction of Beclin1 to Bcl-2. Interaction of Atg14 helps in formation of autophagosome, whereas UVRAG regulates the maturation, fusion, and trafficking stages of autophagy. Binding of Rubicon along with UVRAG inhibits the fusion and trafficking step.

Autophagy is frequently downregulated in various types of tumors suggesting its tumor-suppressive role as discussed earlier [112–114]. Constitutive activation of PI3K-Akt-mTOR axis is known to suppress autophagy [115] and is a characteristic of cancer, by promoting the proliferation, growth, and survival of the tumor cells [116]. There are various proteins like Bcl-2-interacting Beclin1 (BECN1/ATG6), Atg4c [117], Bax-interacting factor-1 (Bif-1) [118], BH3-only proteins [7], DAP kinase [119], ultraviolet radiation resistance-associated gene (UVRAG) [120], and PTEN [121] involved in autophagy that shows its role in tumor suppression (Figure 6). There are also some tumor suppressors like LKB1 [8, 122], TSC [123], nuclear p53 [56], and AMPK [124] which regulate the mTORC pathway. Additionally, all ATGs show this tumor suppressor effect recommending that proteins of autophagy acting at various strides of the pathway have a common tumor suppressor property. Several studies reported that the initiation of autophagy causes radiation sensitization in radio-resistant and malignant glioma cells. Studies have also shown that certain chemotherapeutic drugs may destroy tumor cells through intrinsic pathway of apoptosis initiated by autophagy [125, 126].

*(1) Beclin1 as a Tumor Suppressor*. With the studies on Beclin1, it was first recognised that defective autophagy plays a role in cancer. Beclin1 is an ortholog of yeast Atg6/Vsp30 gene and is required for autophagy and vacuolar protein sorting processes [127]. It maps on the centromeric region of BRCA1 on 17q21 chromosome which is responsible for 75%, 50%, and 40% deletion in many cancers like ovarian, breast, and prostate cancers, respectively [128, 129]. Deletion in both BRCA1 and Beclin1 or only BRCA1 was found, but no proof of Beclin1 mutation or loss detected in any cancer which questions its tumor silencing activity in human cancer. But it is identified as a haploinsufficient tumor suppressor that is deleted monoallelically in cancer and decreases in autophagy [114]. It was the first direct link established between autophagy and cancer. Beclin1^+/−^ immortalized baby mouse kidney (iBMK) cells [106, 110] and immortalized mouse mammary epithelial cells (iMMECs) show compromised autophagy and more tumorigenesis when transplanted *in vivo* in nude mouse allografts [124]. Tissue specificity is seen in the Beclin1 tumor-suppressive function. In contradiction, colorectal and gastric cancer shows higher expression of Beclin1 about 95% and 83%, respectively, compared to the normal stomach and colon mucosa with very low and undetectable levels. The Beclin1 protein contains Bcl-2 homology-3 (BH3) domain which interacts with BH3 receptor domain of antiapoptotic proteins like Bcl-2 and Bcl-xL and inhibits Beclin1 autophagic activity as well as its tumor suppressor activity. BH3-only proteins and DAPK under nutrient deprivation induce autophagy by disrupting Beclin1 interaction to Bcl-2/Bcl-xL competitively [7, 130, 131].

*(2) UVRAG as a Tumor Suppressor*. Reactive oxygen species (ROS), dysfunctional mitochondria, and accumulation of toxic protein aggregates cause aneuploidy and oncogenic transformation in autophagy-deficient cells. Through UVRAG which was isolated in a screen for gene complementing UV sensitivity in xeroderma pigmentosum cells, autophagy can protect against the instability of genome and confer centrosome protection. UVRAG depletion leads to errors in chromosome segregation, malfunctioning of the spindle apparatus [8]. It is represented as a hub for regulating autophagy and cancer as it is a part of Beclin1 and Vps34 multifunctioning protein which behaves as type III PI3K and stimulates autophagy. Low levels of UVRAG cause impaired induction of autophagy. It does not only act early in the initiation of autophagy but also regulates later-on stages like maturation and fusion. Its phosphorylation by mTORC1 blocks the late stages of autophagy. It disrupts Beclin1, dimer stabilized by Bcl-2-like protein to induce autophagy in both *in vivo* and *in vitro*. Similar to Beclin1, it is deleted monoallelically. In colon and gastric cancers, autophagy decreases and it is done by targeting adenine track of UVRAG gene (A10 in exon 8) for frameshift mutation [128, 129].

*(3) Bif-1 as a Tumor Suppressor*. Bif-1/endophilin B1, also known as SH3GLB1, is discovered originally as a Bax-binding protein. It is a third protein associated with the class III PI 3-kinase complex [132, 133]. It participates in vesicle formation and membrane dynamics with membranes of organelles of Golgi apparatus, mitochondria. [118]. During nutrient deprivation, Bif-1 along with LC3/ATG8, ATG5, and ATG9 autophagic effectors gets accumulated in the cytoplasmic puncta. It stimulates PI3K complex activity and regulates autophagy by interacting with Beclin1 through UVRAG, although it does not appear to be a constitutive subunit of the complex. Bif-1 is established as a suppressor, as *bif1^−/−^* mice, and suppresses autophagosome formation and enhances the lymphomas and tumor development [7].

*(4) An ATG Protein as a Tumor Suppressor*. It was reported that *Beclin1^−/−^* mice die early in embryogenesis and aging; mice with *Beclin1^+/−^* are tumor-prone [134, 135], whereas mice with *Atg5^−/−^* and *Atg7^−/−^* are born normally, but die soon due to low nutritional level and suckling defects in neonates [136, 137]. As compared to *Atg5^+/−^* iBMK cells, *Atg5^−/−^* iBMK cells are more tumorigenic in nude mouse allografts [106]. Also, in Atg7-deficient liver, neither cell proliferation nor tumorigenesis was observed [136]. Functions of ATG5 and Beclin1 act as “guardians” of the cellular genome. During ischaemic stress parallel with tumorigenesis, their loss displays gene amplification, aneuploidy, and DNA damage. Also, as compared to ATG5 and ATG7, Beclin1-dependent and Beclin1-independent properties play an important role during embryonic development and tumor suppression. Further autophagy defects playing a role in tumorigenesis are confirmed by Atg4C^−/−^ mice (autophagin-3 cysteine protease involved in the processing of LC3), show decreased autophagy which is starvation induced, and also develop fibrosarcoma by chemical carcinogen induction. Cysteine-specific residues in active sites of protein cause ROS-mediated oxidation due to which Atg4C appears as ROS sensor in autophagy [117, 138]. From the discussion, it is found that autophagy acts as a tumor suppressor and its reduced function is a hallmark of cancer.

#### 4.1.2. Autophagy in Tumor Progression

Autophagy is frequently downregulated in various tumors, but this is not always the case; they are more dependent on autophagy for survival than normal cells/tissues. Several evidences indicate that autophagy maintains tumor cell survival and confers stress tolerance in cancer cell after exposure to various chemo- and radiotherapies and hypoxic and hypothermic conditions [100]. In pancreatic cancer cell lines and tumor specimens, increased basal level of autophagy was detected while inhibition of autophagy leads to tumor regression.

Due to the high proliferation rate of cancer cells, they have high metabolic demand (nutrient, oxygen supplies, etc.). Autophagy provides metabolic substrate to maintain tumor metabolism and helps in the survival of tumor cells under unfavourable conditions. It is reported that knocking down of essential autophagic genes results in impaired survival under metabolic stress which ultimately leads to death in *in vivo* models and various tumor cells [100, 139, 140]. Inhibiting autophagy by genetic or pharmacological means in cancer cells has shown tumor regression. Studies showing mouse survival with ATG7 deficiency in prostrate tumor and intestinal cancer suggest that autophagy helps in tumor progression. Also, in mouse models of Kras-driven glioblastoma, ULK1, Atg7, and Atg13 knockdowns demonstrate that autophagy is crucial for the initiation and sustained growth of glioma. Studies establish that in comparison to ATG7 intact, ATG7 deficiency reduces Kras-driven lung tumor cell proliferation and burden in mice [15]. Poor blood supply or limited nutrient supply to the metastasizing cells to organs has prevalent metabolic stress [141]. In metastasis, autophagy is represented as a mechanism of survival which gets upregulated [142]. For example, deficiency of Atg17/FIP200 inhibits the growth of mammary cancer in mice, suggesting that autophagy has a role in promoting tumorigenesis [143, 144]. In ATG7-deficient mouse, melanoma development, initiation, and proliferation are prevented which prolong survival of mouse. Compared to ATG7-deficient liver, combined p62 and autophagy loss in Atg7^−/−^ liver abrogate inclusion body formation, alleviate hepatic injury, and retard tumor progression [140, 145]. Not only this, p62 accumulation is a hallmark of impaired autophagy. Its overexpression and accumulation in defective autophagy tumor cells is sufficient for ROS and DNA damage response induction under metabolic stress. This causes genetic instability and cell division abnormalities which at the end lead to the progression of tumor [146]. In a study, drug-induced stress in HEp-2 cells relays on autophagy to survive and confer chemoresistance to carcinoma cells by coping with p62-related proteotoxicity [147]. Thus, autophagy is regarded as a mechanism of tumor cell survival and also shows a promising therapeutic target for the treatment of different cancers.

### 4.2. Autophagy: Role in Resistance to Therapy

Autophagy is activated in response to a variety of cancer therapies and may induce cancer cell survival or chemoresistance as an adaptive cellular response [100, 139, 140, 148]. Accumulation of autophagosome has been observed in cancer cells after they were exposed to various chemotherapeutics like temozolomide DNA alkylating agent [149], tamoxifen an estrogen receptor antagonist [150], resveratrol [151], vitamin D_3_, and anthocyanins [152]. Along with these chemotherapeutics, hypoxia, hyperthermia, and radiotherapy activate and upregulate autophagy, thus enabling a dormancy state in the cancerous cells which can lead to tumor reoccurrence and progression [118, 130]. Evidence showed enhanced efficiency of anticancer drugs on tumor cells with inhibited autophagy. Chloroquine inhibits autophagy and also enhances the efficacy of p53 or DNA alkylating agent to induce tumor regression or cell death in c-Myc-induced lymphomas in mice [131]. Through pharmacological means or by knocking down Atgs like Atg5, Atg6, and Atg7 is a major therapeutic means for sensitizing cells to anticancer therapies. Studies have reported that Akt inhibitors like triciribine and perifosine can initiate protective autophagy among tumor cells. Perifosine can also increase the rapamycin cytotoxicity in multiple myeloma by suppressing the PI3K/Akt/mTOR pathway [108]. Furthermore, studies have shown that metformin can suppress progression of tumor by activating AMPK in melanoma and cervical cancer and thus by causing autophagy [153, 154]. Cancer cells can be sensitized to radio- and chemotherapies by siRNA-mediated depletion of ATG proteins, e.g., miRNA-22 is shown to influence chemotherapy-resistant colon cancer cells by inhibiting autophagy and promoting apoptosis [111, 155]. Overexpression of miR-22 in colon cancer cells increased sensitivity to 5-FU which is one of the main chemotherapeutic agents used in the treatment of colorectal cancer [156]. miR-409-3p inhibits autophagy by targeting Beclin-1 resulting in enhanced sensitivity to oxaliplatin. miRNA-210 induces autophagy and reduces radiosensitivity in colon cancer [58]. In a study, miR137 chemosensitize pancreatic cancer cells and inhibit autophagy by targeting Atg5 [157]. It has also been seen that long noncoding RNAs (lncRNAs) and CAIF (cardiac autophagy inhibitory factor) modulate autophagy and prevent defective autophagy-mediated loss of cardiac myocytes. It could be a potential therapeutic tool for the treatment of myocardial infarction and heart failure [158]. Other noncoding RNAs like circular RNA (Hsa-circ0023404) have a role in cervical cancer progression, metastasis, and chemoresistance through autophagy by sponging miR5047 [159]. Thus, it is clear that miRNA and any other noncoding RNAs regulate autophagy under various stress conditions. Various strategies are available to deliver the knockout materials (miRNAs or siRNA) to the target cancer cells such as nanoparticles, lipid vectors, nonlipid vectors, and multistage vector (MSV) delivery system. Lipid vectors such as liposomes and stable nucleic acid lipid particles (SNALPs) and nonlipid vectors such as chitosan, poly(amidoamine) dendrimers, and polyethylenimines are used [160]. Cyclodextrin nanoparticles can be used as an effective tumor targeting, as the surface is decorated with transferrin protein targeting ligand since the transferrin receptors are overexpressed on the surface of cancer cells [161]. Radiosensitization of malignant glioma cells can also be caused by using autophagy inhibitors like 3-methyladenine and bafilomycin A1 [162].

#### 4.2.1. Pharmacological Modulation of Autophagy to Counteract Cancer

Activators of autophagy are considered effective in neurodegenerative diseases, whereas inhibitors of autophagic flux such as chloroquine are considered effective in cancer therapy. It is a fact that autophagy in normal cells is beneficial and the use of its inhibitors as cancer therapy is a major obstacle [8]. So, a drug that targets the autophagic pathway in cancer cells without affecting normal cells could be an ideal drug. This novel paradigm in cancer therapy has been validated in several preclinical studies and is now under investigation in I/II phase of clinical trials involving autophagy inhibition. Antimalarial drug hydroxychloroquine, which blocks lysosomal degradation of the autophagy products by affecting lysosomal pH, in combination with anticancerous drugs involved in standard chemotherapy to achieve better outcomes.  Table 1 shows a few of the clinical trials based on the antimalarial drugs in combination with different anticancer drugs used for the inhibition of autophagy. They support that targeting autophagy provides therapeutic benefits in models of chemotherapy resistance and shows very promising results in combinatorial cancer treatment.

Apart from inhibition of autophagy as a tumor-suppressive mechanism in the early stages of tumor formation, there may be a benefit in identifying activators of autophagy as anticancer agents as well. Significant attempts are being devoted to the development of autophagy modulator agents with enhanced pharmacological specificity. Activators such as IFN*γ*, melatonin, and trehalose and inhibitors such as LY294002 are in clinical trials, whereas activators such as A-769662, BECN1-derived peptide, and BRD5631 and inhibitors such as compound C (also known as dorsomorphin), Mdivi-1, Lys05, SAR405, VPS34-IN1, SBI-0206965, MRT67307, NSC185058, and MRT68921 are in preclinical development stage [163]. There is little evidence that induction of autophagic cell deaths can also be used as a therapeutic strategy for removing cells with high apoptotic threshold or compromised apoptosis cancerous cells lacking BAK, BAX, and caspases. For instance, thalidezine in various cancer cell lines eliminates apoptotic resistance via autophagic cell death [164]; four dauricine derivatives induce autophagy-dependent cell death in HeLa cells [165]. Some natural products in in vivo studies like polyphyllin (PPI) extracted from rhizome *Paris polyphylla* activate AMPK directly and suppress the growth of non-small-cell lung cancer (NSLC) [166]. Ethoxysanguinarine, an alkaloid extracted from *Macleaya cordata*, induces autophagy cell death in breast cancer cells [167]. Various rapamycin derivatives such as CCI-799, RAD001, and AP23573 can also be used as effective therapeutic agents against cancer [168]. In combination with chemotherapeutic agents, autophagic cell death is also found in *in vitro* cancer cells. In MCF-7, the silencing of Bcl-2 by siRNA leads to autophagic cell death. Further siRNA in combination with a low dose of doxorubicin enhances autophagy which inhibits tumor growth and leads to autophagic cell death [169].

Various potential drugs and their nanoformulations such as Doxil (liposomal formulation of doxorubicin) which is a first approved nanodrug used in breast cancer therapy and Abraxane (albumin-bound paclitaxel formulation) can be used in combinational strategies to improve therapeutic effect and reduce the toxicity and side effects [160]. But after all this, future studies are needed to prove that manipulation of autophagy can be useful in clinics.

### 4.3. Autophagy and Immune System

**Figure 7:**
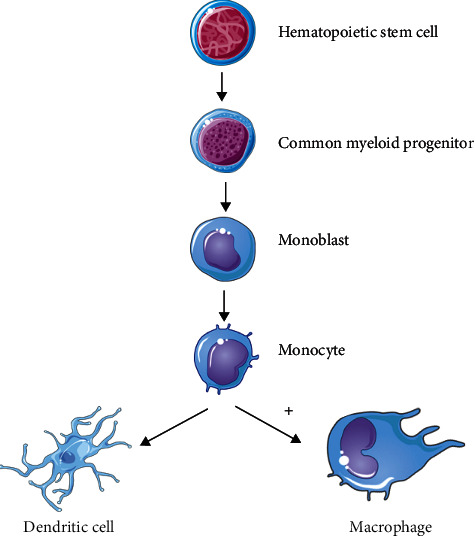
Autophagic activity in differentiation from monocytes to macrophages. Immune cells like dendritic cells and macrophages arise from hematopoietic cell (HSC) which has dedicated to common myeloid progenitor. Monoblast then generates a monocytic cell line which leads to the development of dendritic cells and macrophages. Autophagy involves in the differentiation process of monocytes to macrophages. “+”: enhanced function.

Autophagy is an important catabolic pathway which plays several roles in different kinds of cells [170]. Autophagy is also necessary for complete macrophage differentiation of mice and human monocytes driven by granulocyte-macrophage colony-stimulating factor (GM-CSF) or colony-stimulating factor-1 (CSF-1) [171]. The initiation of autophagy is crucial for differentiation and survival of monocytes. The signal of differentiation discharges Bcl-2 interacting coiled coil protein-1 (Beclin-1) from Bcl-2 by c-Jun N-terminal kinase (JNK) activation and preventing cleavage of ATG5 which are important for induction of autophagy [172]. Prevention of autophagy initiation hinders production of cytokines and differentiation. Hence, autophagy is vital in the conversion from apoptosis of monocyte to differentiation (Figure 7) [172]. In innate immune system, autophagy also modifies cell-specific functions like phagocytosis and antigen presentation. Autophagy can also play a role in microbial defence like antimicrobial peptide production and antigen presentation as pathogens/microbes can be engulfed directly in autophagolysosomes [170].

## 5. Conclusion and Future Perspective

Autophagy is a cell's recycling machinery, degrades cytosolic substrates in lysosomes, and provides by-products (biomolecules) which are used in anabolic processes and help cells survive in stress conditions. It is under tight regulation of mTOR, various transcriptional factors, p53 localization, etc. Thus, autophagy plays an important role not only in normal cells but also in cancer cell progression. It acts as a double-edged sword; on the one hand, it promotes tumor survival by providing energy and maintains homeostasis, whereas on the other hand, its defects elevate oxidative stress, damage, and mutations which are linked to tumor initiation and progression. It acts as a tumor suppressor during initial stages of cancer but becomes a tumor progressor in advanced stages. It is also responsible for providing resistance against various cancer therapies. Blocking autophagy is an approach to treat established, aggressive cancer, providing a promising hope for clinical applications. Inhibition of autophagy by pharmacological means or by ATG knockdown can promote cancer cell death and could be therapeutically advantageous but only without affecting normal cells. Other than autophagy regression, its contradictory role, i.e., tumor suppression at the early stages of cancer development, should also be considered. More effort has to be put on the development of effective cancer-specific delivery systems and drugs to make tumor more sensitized to therapies. In addition, a detailed deciphering of the crucial role of noncoding RNAs in autophagy has profound clinical implications. Therefore, along with combinational therapies with the conventional one, more effort should be put on the miRNA-based therapies for therapy-resistant cancers.

The role of autophagy in the development and maintenance of cancer is thoroughly studied, but still there are many aspects of this that need to be addressed. The cancer cells maintain higher basal level autophagy which supports their survival even under various hypoxic conditions and its regulation mechanism is still not fully understood. Also, enlightenment on the mechanism of cargo selection for this heightened basal level autophagy in cancer cells has not been elucidated. Many autophagy-related genes are studied which play an important part in protection and development of therapeutic resistance in various cancer cells, but their regulation and molecular mechanisms are yet to be deciphered fully. The scope of understanding tumor microenvironment is getting attention since tumors can develop and proliferate in various stressful hypoxic and hypoglycemic conditions. This characteristic of cancer is mainly attributed to increased autophagy in cancerous cells. Thus, understanding the interaction between tumor microenvironment and autophagy is of utmost importance which involves various *in vivo* as well as organotypic culture studies. These aspects of autophagy can be highly advantageous in developing various therapeutic regimens which can impede tumor development and progression as well as countering therapeutic resistance. Last but not the least, a lot of effort is still needed to understand the types of various cancers that would be controlled using autophagy inhibition which will depend on the development of newer and better biomarkers of autophagy in cancer.

## Figures and Tables

**Table 1:** Antimalarial drug combinations are used for cancer treatment in various preclinical studies [173–175].

Compound (autophagy inhibitor)	Indication	Phase	Trial ref # at ClinicalTrials.gov
Chloroquine (CQ)	Small-cell lung cancer	I	NCT00969306
	Breast cancer	II	NCT02333890
Chloroquine + tamoxifen	Breast ductal carcinoma	II	NCT01023477
Velcade and cyclophosphamide with CQ	Multiple myeloma	II	NCT01438177
Cisplatin and etoposide + CQ	Stage 4 small-cell lung cancer	I	NCT00969306
Hydroxychloroquine (combination treatment)	Non-small-cell lung cancer	II	NCT00933803
	Colorectal cancer	II	NCT01006369
	Advanced cancer	I	NCT01266057
	Rectal cancer, colon cancer, metastasis, adenocarcinoma	II	NCT01206530
Hydroxychloroquine	Melanoma (skin)	I	NCT00962845
	Renal cell carcinoma	I	NCT01144169
	Unspecified adult solid tumor	I	NCT00909831
Hydroxychloroquine + ixabepilone	Breast cancer	II	NCT00765765
Hydroxychloroquine + docetaxel	Prostate cancer	II	NCT00786682
Hydroxychloroquine + vorinostat	Advanced solid tumor	I	NCT01023737
Hydroxychloroquine (combination treatment)	Lung cancer	II	NCT00728845
Hydroxychloroquine + bortezomib	Multiple myeloma and plasma cell neoplasm	II	NCT00568880
Hydroxychloroquine + gemcitabine	Pancreatic cancer	II	NCT01128296
Gemcitabine and Abraxane with or without HCQ		II	NCT01978184
		II	NCT01506973
